# Cardiovascular Disease Mortality and Its Association With Socioeconomic Status: Findings From a Population-based Cohort Study in Rural Vietnam, 1999–2003

**Published:** 2006-06-15

**Authors:** Hoang Van Minh, Dao Lan Huong, Stig Wall, Peter Byass, Nguyen Thi Kim Chuc

**Affiliations:** Hanoi Medical University; Health Strategy and Policy Institute, Ministry of Health, Hanoi, Vietnam; Umeå International School of Public Health, Umeå University, Umeå, Sweden; Umeå International School of Public Health, Umeå University, Umeå, Sweden; Hanoi Medical University, Hanoi, Vietnam

## Abstract

**Introduction:**

Cardiovascular disease is an emerging epidemic in Vietnam, but because cause of death and other routine data are not widely available, it is difficult to characterize community-based disease patterns. Using 5-year data from an ongoing cause-specific mortality study conducted within a demographic surveillance system in Vietnam's Bavi district, this article estimates the rates of adult cardiovascular disease mortality in relation to the mortality rates of other noncommunicable diseases in rural northern Vietnam and examines the association of cardiovascular disease with certain demographic and socioeconomic factors.

**Methods:**

All causes of death of adults aged 20 and older occurring from 1999 through 2003 (n = 1067) were determined by using an established demographic surveillance system and data collected by trained interviewers who asked caretakers or relatives of the deceased individuals about signs and symptoms of disease during quarterly household visits. Deaths were classified as cardiovascular disease, cancer, or other noncommunicable diseases. These records were linked to demographic and socioeconomic data.

**Results:**

Of the 1067 adult deaths that were recorded, there was an overall noncommunicable disease mortality rate of 7.8 per 1000 person-years. Cardiovascular disease accounted for 33% of male and 31% of female deaths. Compared with cancer and other noncommunicable causes of death in a Cox proportional hazards model, higher cardiovascular disease mortality rates were observed among men, older age groups, and those without formal education.

**Conclusion:**

To date, cohort studies and population-based mortality data in Vietnam have been scarce; this study provides insights into the public health aspects of cardiovascular disease in transitional Vietnam. The rates of cardiovascular disease mortality in this rural Vietnamese community were high, suggesting the need for both primary prevention and secondary treatment initiatives. The demographic surveillance system is an important tool for characterizing such an epidemic.

## Introduction

Emerging epidemics of cardiovascular disease (CVD) have attracted attention as major causes of global disability and mortality (1,2). In 1990, it was estimated that 15 of 52 million deaths worldwide were attributable to CVD, and 63% of those deaths occurred in developing countries (2). CVD (mainly heart disease and stroke) was responsible for approximately half of noncommunicable disease (NCD) mortality and one quarter of the NCD morbidity rate in 1999; low- and middle-income countries were most affected (3). Ischemic heart disease and stroke are projected to be first- and fourth-ranking contributors to global disability adjusted life-years lost by 2020, and developing countries will experience most of the increase (2).

Like other developing countries, Vietnam is undergoing a health transition characterized by increasing NCDs and dominated by CVD (4). Hospital statistics for Vietnam show that NCDs constituted 39% of admissions in 1986 and 65% in 1997, and NCD mortality rose from 42% in 1986 to 62% in 1997 (5). By 1998, hospital deaths from CVD were commonly reported; among all causes of death, stroke ranked first, acute myocardial infarction ranked fourth, hypertension ranked fifth, and heart failure ranked seventh (6). In 2002, intracerebral hemorrhage, hypertension, and heart failure were among the five leading causes of morbidity and mortality in hospitals (7).

Hospital-based data can reflect only part of disease patterns; it is also important to understand changing patterns of NCD and CVD in the community to fully understand emerging epidemics. Because national community-based data are lacking in Vietnam, population surveillance approaches need to be used.

Using 5-year data from an ongoing cause-specific mortality study conducted within a demographic surveillance system (DSS) in the Bavi district, this article estimates the burden of adult CVD mortality in relation to other NCD mortality in northern Vietnam and examines its association with demographic and socioeconomic factors. Bavi district is a predominantly rural area in which most people rely on agricultural production. Health services are provided through a district health center in Bavi in addition to commune health stations and a few private providers. A DSS offers the opportunity to identify all deaths in a community, retrospectively determine causes of death, and make links to prospectively gathered background factors such as educational and socioeconomic status (8).

To date, cohort studies and population-based mortality data in Vietnam have been scarce; this study provides insights into the emerging epidemic of CVD in Vietnam. Knowing the extent of CVD mortality, as well as its demographic and socioeconomic determinants, is essential for primary prevention strategies and effective case detection and clinical management.

## Methods

### Study setting

This cause-specific mortality study was carried out in the Bavi district epidemiological field laboratory (FilaBavi), a DSS in which the original sample was selected randomly with probability proportional to population and which covers a range of geographical regions in the district. The sampling unit was hamlet or village subdivision, or *cluster*; it included 67 clusters with an estimated 11,300 households and a total population of about 51,000. The methods of this study have been published elsewhere (9).

### Data collection

All cases of death among people aged 20 years and older that occurred from January 1, 1999 through December 31, 2003 (n = 1067) were captured for analysis by death registration obtained during quarterly household visits. A *verbal autopsy* questionnaire, based on a World Health Organization (WHO) instrument and adapted for Vietnam, was administered by trained interviewers. Interviewers assigned the most likely cause of death by obtaining information from a close relative or caretaker of a deceased person about circumstances, signs, and symptoms during the illness (10).  When the verbal autopsy questionnaire had been completed, it was forwarded to two physicians who undertook the interpretation and diagnosis procedure for the data. They each made independent diagnoses based on the reported circumstances, signs, and symptoms. In cases in which the physicians differed, they held discussions to reach consensus. A Kappa test was used to assess degree of agreement between the two physicians. NCD deaths were classified as CVD, cancer, or other NCDs.

### Data analysis

Person-time generated from the dynamic cohort of people under surveillance during the same period were calculated based on the date of events (i.e., in-migration, out-migration, births, and death) for age- and sex-specific groups and used as denominators (8). Age was first categorized into the following three groups: 20 to 49 years (young adults), 50 to 74 years (mid-adults), and 75 years or older (elderly). To provide more significant results, age was reclassified into the following two groups: 20 to 49 years and 50 years or older.

Individual socioeconomic status was estimated by assessing education level and household economic status prospectively at the time of entering the cohort in the DSS. Education level was classified as either no formal education (including illiteracy) or formal education (completion of any level of schooling). Economic condition of households was described as either poor (average monthly income per person less than VND 45,000 or U.S. $3.30) or nonpoor (average monthly income per person more than or equal to VND 45,000 or U.S. $3.30) (People Committee of Bavi: Decision number 59, Ministry of Labor, Invalids and Society, adapted for Bavi district, 1999).

Analyses were carried out using Stata software, version 8 (Stata Corp, College Station, Tex). CVD mortality rates per 1000 person-years were calculated. Crude and adjusted Mantel-Haenszel rate ratios and corresponding 95% confidence intervals were used to compare mortality rates by sex. Multivariate Cox proportional hazards models were used to examine associations between sex, age, socioeconomic status, and CVD mortality. The effect of each independent variable was assessed by examining relative risk while controlling for other independent variables in the model. To take into account the nonrandom sampling nature of verbal autopsy, cluster analysis was introduced on the calculation and modeling.

The surveyed population is described in Table 1 by sex, age, education, and economic status. There were 14,289 men and 16,713 women aged 20 years and older at the beginning of the study period. The proportion of people without formal education was higher among women (18%) than men (5%). About 12% of men and 14% of women were classified as poor.

## Results

During the 5-year study, there were a total of 1067 deaths (572 among men and 495 among women) occurring among 137,172 person-years within the cohort (data not shown). Thus, the overall mortality rate for all causes of death in the study sample was 7.8 per 1000 person-years (9.2 among men and 6.6 among women).

Table 2 shows that NCDs accounted for a substantial proportion of total mortality (67% of the men and 61% of the women). CVD was the most common cause of death and accounted for 33% of deaths among men and 31% among women and was the largest component of NCD mortality. Deaths from cancer comprised 17% of deaths among men and 14% of deaths among women. Other NCDs (e.g., chronic obstructive lung disease, kidney failure, cirrhosis) were responsible for 17% of deaths among men and 16% among women. Among CVD deaths, stroke was the predominant cause (59% of men and 64% of women). Heart failure was the second-ranked CVD cause of death (19% among both men and women), and the remaining CVD causes of death were coronary heart disease, pulmonary heart disease, mitral and aortic valve disease, and others (21% of men and 17% of women).

A graphical representation of cumulative adult mortality rates for three subgroups of NCD (CVD, cancer, and other NCDs) and all other causes of death by sex is presented in the Figure. Mortality rates from NCDs and all other causes of death increased consistently with age and were higher for men than women. Changes with age and sex differences were most notable for CVD.

**Figure:**
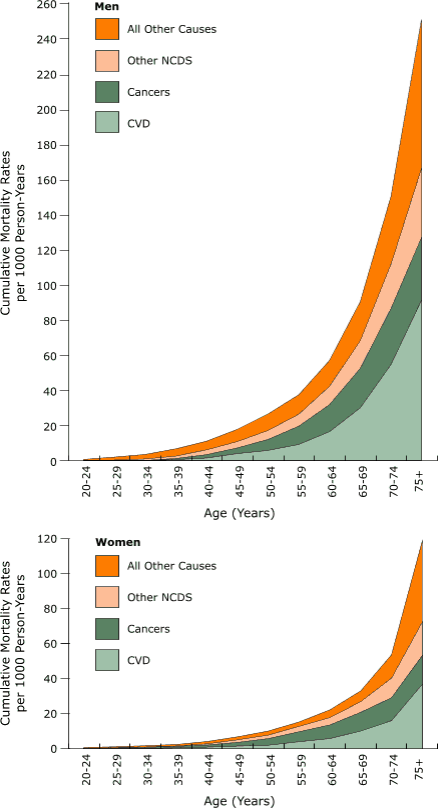
Cumulative mortality rates for NCDs among men and women aged 20 years and older, Bavi district, Vietnam, 1999–2003.

**Table:**

Table 3 shows NCD mortality rates per 1000 person-years by sex, age, education, and economic status. Sex rate ratios are presented in Table 4. CVD mortality rates (3.0 for men and 2.1 for women) were higher than those for cancer and other NCDs. NCD mortality rates increased with age and decreased with education, an effect that was strongest for CVD. Mantel-Haenszel combined rate ratios showed significantly greater mortality risks for men in all three NCD subgroups, particularly in relation to education.

The results of multivariate Cox proportional hazards modeling of mortality risk factors are shown in Table 5. After controlling for other independent variables, the significant predictors of NCD mortality in the study population were male sex, age of 50 years or older, and lack of formal education. The model also showed that CVD mortality was more strongly associated with sex, age, and education than other NCD mortality causes. Sex-specific Cox models confirmed similar effects of age and education for men and women separately (data not shown).

Joint effects of age and education on CVD mortality were further examined and are presented in Table 6. Compared with people aged 20 to 49 years old with formal education, the risk of dying from CVD was 7 times higher in the same age group with no formal education, 14 times higher among those aged 50 years and older with formal education, and 61 times higher for those aged 50 years and older with no formal education.

## Discussion

Cohort studies and population-based mortality data in Vietnam have been scarce, and there has been little knowledge of the magnitude of the burden of CVD mortality and its association with socioeconomic status. This study, conducted within the framework of a well-functioning DSS uniquely positioned for assessing epidemiological transition (8), provides insights into the public health aspects of CVD in transitional Vietnam.

### Burden of CVD mortality and stage of CVD epidemic

We have shown that a substantial proportion of deaths are attributable to CVD, which was the leading cause of death among adults. The substantial effects of CVD mortality can be partly explained by the aging of the population in the setting. The proportion of people aged 50 years and older rose from 16.1% (13.4% of men and 18.7% of women) in 1999 to 16.9% (14.2% of men and 19.5% of women) in 2001 and to 17.9% (15.0% of men and 20.5% of women) in 2003. 

Epidemiological transition pertaining to CVD epidemics has classically been described in four stages (11). In stage one, the predominant circulatory diseases are rheumatic heart disease, those attributable to other infections, and nutritional deficiency-related disorders of the heart muscle. In the second stage, as infectious disease rates decline and nutrition condition improves, diseases related to hypertension, such as hemorrhagic stroke and hypertensive heart disease, become more common. In the third stage, which includes the highest CVD mortality rates, atherosclerotic processes lead to a high incidence of ischemic heart disease and atherothrombotic stroke, especially at ages older than 50 years. During the fourth stage, increased efforts to prevent, diagnose, and treat ischemic heart disease and stroke typically delay these diseases until older ages.

Our results suggest that rural Vietnam, as represented by the Bavi district, is in the second stage of the CVD epidemic, as are China and some other Asian countries. India is already progressing to stage three of the CVD epidemic, whereas developed countries are typically in stage four (11).

### Effect of sex, age, and education on CVD mortality

Of particular interest in this article are differences in CVD mortality by demographic and socioeconomic conditions because, according to transition theory (12), subsets of populations may be at different stages of the CVD epidemic.

Our results show that CVD mortality rates were significantly higher for men than women, and the differences in mortality by sex were larger for CVD than for other NCD causes. The greater risk for men of dying from CVD was also stated by the American Heart Association (13) and can be explained by the differences in risk factor profiles between men and women. In our setting, men smoked more and had a higher prevalence of elevated blood pressure (14,15). In this study, age was proven to be more strongly associated with CVD mortality than other NCD causes.

We found that CVD mortality rates decreased considerably among educated people compared with those without formal education, even after adjusting for other independent variables such as sex, age, and economic status. This finding is similar to the findings of numerous studies that showed an inverse socioeconomic gradient in CVD mortality in developed (15-18) and developing countries (19).

Likewise, education was an important factor for health among men and among women, particularly in rural areas, because we found that education is usually associated with increased knowledge about health matters and consequent reduction in risky health behaviors. Explanations for the differences in education levels we observed among those who died from CVD include differences in risk factors such as blood pressure, blood cholesterol, smoking, and obesity. Indeed, available evidence from other studies in the Bavi district support the observation that people with lower education levels smoke more (20) and have more hypertension (14). 

In this study, CVD mortality was found to have stronger association with education than other NCD mortality in both sexes. This finding is similar to findings from studies in England (21), Israel (22), and Korea (23).  

A strong correlation between old age (50 years and older) and education was also found in relation to CVD mortality. Even though the risk of dying from CVD associated with lower education level was similar for men and women, the effects of women's education are particularly noteworthy. Given the results, together with the relatively higher proportion of women aged 50 years and older (30% of women aged 20 years and older), of whom 55% had no formal education, better education for women may substantially reduce CVD mortality. Better education likely leads to healthier lifestyles (e.g., lower rates of smoking, drinking, physical inactivity) and is likely to improve access to health care.

Economic status was not significantly associated with CVD mortality in the multivariate regression model. This model showed a possibly rising burden of CVD mortality among the poor, which demonstrates the shift from stage one to stage two of a CVD epidemic.

### Methodological considerations

Because we were working in a setting without medical death certification, our findings depend on the quality of the verbal autopsies. There are numerous factors that may affect the validity of verbal autopsies, including cause of death, characteristics of the deceased individuals, classification of causes of death, the design and content of the questionnaire, and interviewing procedures. The verbal autopsy process is difficult to validate rigorously, particularly for detailed differentiation between different types of CVD. Based on experiences with verbal autopsy in 1999 (24), we chose interviewers with more appropriate qualifications (e.g., medical background, field experience), carefully organized the training, designed a detailed questionnaire, and involved one local clinical physician as an assessor of the questionnaire instrument. Because of the lack of availability of medical documents and records stored either by the family or in health facilities, reliable standards such as health facility or hospital diagnoses and postmortems were not available. However, in settings with poor vital registration and weak health systems, especially at the community level, the verbal autopsy method is the only viable option for determining cause of death (24). We are currently working on the development of a probabilistic model for interpreting verbal autopsy, which may lead to a more objective assessment of cause of death (25).

In this study, education was used as a main indicator of socioeconomic position, which has several advantages. It occurs causally before occupation and income and is stable throughout life after young adulthood. Unlike occupational class, education allows classification of individuals who do not work, including, for instance, most of the elderly individuals in our study. Educational status is an individual measure of socioeconomic position, which may be a better indicator than household measures, such as household income, which are difficult to measure in our setting (26).

Economic status is a household-level indicator, can change over time, and is difficult to measure correctly in our context (26). It may, however, provide another dimension of the relationship between socioeconomic status and CVD mortality.

### Policy implications and suggestions for further studies

In conclusion, this study demonstrated a heavy burden of CVD mortality among the adult population in a rural community of Vietnam, during a period of epidemiological transition. It also suggests that education may be an important factor in preventing deaths from NCD and CVD, especially in reducing CVD mortality among women. This study also demonstrates the potential of the DSS as a means of characterizing a major community-based epidemic in a setting where routine data collection is insufficient for effective health policy making and planning.

There is an urgent need to develop and implement effective CVD policies and interventions in Vietnam. Interventions should include both primary prevention, (e.g., educating people about health hazards, promoting healthy lifestyles) and secondary approaches (e.g., improving geriatric services). Whether adult education programs can offer any prevention against CVD remains an open question. Further studies over longer periods are required to give more insight into the CVD epidemic. The link between CVD risk factors and mortality in transitional settings also needs further investigation to prioritize health promotion messages.

## Figures and Tables

**Table 1:** Population Cohort Characteristics, Bavi District, Vietnam, 1999–2003

**Variable**	**Men No. (%)**	**Women No. (%)**	**All No. (%)**
**Total**	**14,289 (100)**	**16,713 (100)**	**31,002 (100)**
Age, y			
20–49	10,985 (76.9)	11,670 (69.8)	22,655 (73.1)
50–74	2,884 (20.2)	3,946 (23.6)	6,830 (22.0)
≥75	420 (2.9)	1,097 (6.6)	1,517 (4.9)
Education			
No formal education	699 (4.9)	3012 (18.0)	3,711 (12.0)
Formal education^a^	13,590 (95.1)	13,701 (82.0)	27,291 (88.0)
Economic status^b^			
Poor	1,624 (11.8)	2,278 (14.1)	3,902 (13.0)
Nonpoor	12,174 (88.2)	13,871 (85.9)	26,045 (87.0)

a Formal education was defined as having attended any primary school or more.

b Poor economic status was defined as average monthly income per person less than VND 45,000 or U.S. $3.3; nonpoor economic status was defined as average monthly income per person greater than or equal to VND 45,000 or U.S. $3.3. (Decision number 59, Ministry of Labor, Invalids and Society, adapted for Bavi district)

**Table 2:** Causes of Death Among Adults Aged 20 Years and Older, Bavi District, Vietnam, 1999–2003

**Causes of Death**	**Men No. (%)**	**Women No. (%)**	**All No. (%)**
**Total**	**572 (100)**	**495 (100)**	**1067 (100)**
All NCDs	381 (67)	301 (61)	682 (64)
Cardiovascular diseases	190 (33)	154 (31)	344 (32)
Stroke	113 (59)	99 (64)	212 (62)
Heart failure	37 (19)	29 (19)	66 (19)
Other	40 (21)	26 (17)	66 (19)
Cancer	95 (17)	70 (14)	165 (16)
Other NCDs	96 (17)	77 (16)	173 (16)
Other	191 (33)	194 (39)	385 (36)

NCDs indicates noncommunicable diseases.

**Table 3:** Mortality Rates for Cardiovascular Disease (CVD), Cancer, and Other Noncommunicable Diseases (NCDs) Among Adults Aged 20 Years and Older (n = 1067), Bavi District, Vietnam, 1999–2003

**Variable**	**Men Rate per 1000 Person-Years (95% CI)**			**Women Rate per 1000 Person-Years (95% CI)**		
	**CVD**	**Cancer**	**Other NCDs**	**CVD**	**Cancer**	**Other NCDs**
**Overall**	**3.0(2.6-3.5)**	**1.5(1.2-1.9)**	**1.5(1.3-1.9)**	**2.1(1.8-2.4)**	**0.9(0.7-1.2)**	**1.0(0.8-1.3)**
Age, y						
20–49	0.5 (0.4-0.8)	0.4 (0.3-0.7)	0.5 (0.4-0.8)	0.2 (0.1-0.4)	0.3 (0.2-0.5)	0.2 (0.1-0.4)
≥50	11.2 (9.7-13.1)	5.0 (4.0-6.3)	4.8 (3.8-6.0)	6.3 (5.3-7.4)	2.4 (1.8-3.1)	3.0 (2.4-3.8)
Education						
No formal education	23.1 (18.1-29.4)	6.3 (4.0-10.0)	7.7 (5.1-11.7)	10 (8.4-11.8)	2.8 (2.1-3.9)	3.5 (2.6-4.7)
Formal education^a^	2.1 (1.7-2.5)	1.3 (1.0-1.6)	1.2 (1.0-1.6)	0.4 (0.3-0.6)	0.5 (0.4-0.8)	0.5 (0.4-0.7)
Economic status^b^						
Poor	2.8 (1.8-4.3)	1.7 (0.9-2.9)	2.5 (1.6-4.0)	2.0 (1.3-3.1)	1.2 (0.7-2.0)	1.1 (0.6-1.9)
Nonpoor	2.9 (2.5-3.4)	1.4 (1.1-1.8)	1.4 (1.1-1.7)	1.9 (1.6-2.3)	0.8 (0.6-1.0)	0.9 (0.7-1.2)

CI indicates confidence interval.

a Formal education was defined as having attended any primary school or more.

b Poor economic status was defined as average monthly income per person less than VND 45,000 or U.S. $3.3; nonpoor economic status was defined as average monthly income per person greater than or equal to VND 45,000 or U.S. $3.3. (Decision number 59, Ministry of Labor, Invalids and Society, adapted for Bavi district)

**Table 4:** Sex Rate Ratios for Cardiovascular Disease (CVD), Cancer, and Other Noncommunicable Diseases (NCDs) Among Adults Aged 20 Years and Older (n = 1067), Bavi District, Vietnam, 1999–2003

**Variable**	**CVD Men:Women Rate Ratio (95% CI)**	**Cancer Men:Women Rate Ratio (95% CI)**	**Other NCDs Men:Women Rate Ratio (95% CI)**
**Overall**	**1.5 (1.2-1.8)**	**1.6 (1.2-2.2)**	**1.5 (1.1-2.0)**
Age, y			
20–49	2.1 (1.0-4.5)	1.4 (0.7-2.9)	2.8 (1.3-6.6)
≥50	1.8 (1.4-2.3)	2.1 (1.5-3.0)	1.6 (1.1-2.3)
Mantel-Haenszel combined rate ratio	1.8 (1.5-2.2)	1.7 (1.4-2.6)	1.8 (1.3-2.4)
Education			
No formal education	2.3 (1.7-3.1)	2.2 (1.2-4.0)	2.2 (1.3-3.7)
Formal education^a^	5.3 (3.4-8.6)	2.4 (1.6-3.7)	2.5 (1.6-3.9)
Mantel-Haenszel combined rate ratio	3.3 (2.6-4.3)	2.4 (1.7-3.3)	2.4 (1.7-3.3)
Economic status^b^			
Poor	1.4 (0.7-2.7)	1.4 (0.6-3.5)	2.4 (1.1-5.5)
Nonpoor	1.5 (1.2-2.0)	1.8 (1.3-2.7)	1.5 (1.1-2.2)
Mantel-Haenszel combined rate ratio	1.5 (1.2-1.9)	1.8 (1.3-2.5)	1.6 (1.2-2.2)

CI indicates confidence interval.

a Formal education was defined as having attended any primary school or more.

b Poor economic status was defined as average monthly income per person less than VND 45,000 or U.S. $3.3; nonpoor economic status was defined as average monthly income per person greater than or equal to VND 45,000 or U.S. $3.3. (Decision number 59, Ministry of Labor, Invalids and Society, adapted for Bavi district)

**Table 5:** Risk Ratios for Cardiovascular Disease (CVD), Cancer, and Other Noncommunicable Diseases (NCDs) Among Adults Aged 20 Years and Older (n = 1067), Bavi District, Vietnam, 1999–2003

**Variable**	**CVD Risk Ratio (95% CI)**	**Cancer Risk Ratio (95% CI)**	**Other NCDs Risk Ratio (95% CI)**
Sex			
Female	Ref	Ref	Ref
Male	3.3 (2.6-4.3)	2.4 (1.7-3.4)	2.5 (1.8-3.6)
Age, y			
20–49	Ref	Ref	Ref
≥50	13.3 (9.1-19.4)	7.8 (5.2-11.7)	9.7 (6.3-14.9)
Education			
Formal education^a^	Ref	Ref	Ref
No formal education	4.5 (3.4-5.8)	1.5 (1.0-2.3)	2.0 (1.3-2.9)
Economic status^b^			
Poor	Ref	Ref	Ref
Nonpoor	1.1 (0.8-1.6)	0.7 (0.5-1.1)	0.7 (0.4-1.0)

CI indicates confidence interval; ref, reference group.

a Formal education was defined as having attended any primary school or more.

b Poor economic status was defined as average monthly income per person less than VND 45,000 or U.S. $3.3; nonpoor economic status was defined as average monthly income per person greater than or equal to VND 45,000 or U.S. $3.3. (Decision number 59, Ministry of Labor, Invalids and Society, adapted for Bavi district)

**Table 6:** Relative Risk of Age and Education on Cardiovascular Disease Mortality Among People Aged 20 Years and Older, Bavi District, Vietnam, 1999–2003

**Education Level**	**Aged 20-49 y**	**Aged ≥50 y**
No formal education	7.2	60.8
Formal education^a^	Ref	13.8

Ref indicates reference group.

a Formal education was defined as having attended any primary school or more.
